# Impaired Kallikrein-Kinin System in COVID-19 Patients' Severity

**DOI:** 10.3389/fimmu.2022.909342

**Published:** 2022-06-22

**Authors:** Enrique Alfaro, Elena Díaz-García, Sara García-Tovar, Ester Zamarrón, Alberto Mangas, Raúl Galera, Kapil Nanwani-Nanwani, Rebeca Pérez-de-Diego, Eduardo López-Collazo, Francisco García-Río, Carolina Cubillos-Zapata

**Affiliations:** ^1^ Respiratory Diseases Group, Respiratory Service, La Paz University Hospital, IdiPAZ, Madrid, Spain; ^2^ Biomedical Research Networking Center on Respiratory Diseases (CIBERES), Madrid, Spain; ^3^ Department of Intensive Medicine, La Paz University Hospital, Madrid, Spain; ^4^ Laboratory of Immunogenetics of Human Diseases, La Paz University Hospital, IdiPAZ, Madrid, Spain; ^5^ Interdepartmental Group of Immunodeficiencies, Madrid, Spain; ^6^ The Innate Immune Response Group, IdiPAZ, La Paz University Hospital, Madrid, Spain; ^7^ Faculty of Medicine, Autonomous University of Madrid, Madrid, Spain

**Keywords:** bradykinin (BK), COVID-19, inflammation, thromboinflammation, NLRP3 inflammasome

## Abstract

COVID-19 has emerged as a devastating disease in the last 2 years. Many authors appointed to the importance of kallikrein-kinin system (KKS) in COVID-19 pathophysiology as it is involved in inflammation, vascular homeostasis, and coagulation. We aim to study the bradykinin cascade and its involvement in severity of patients with COVID-19. This is an observational cohort study involving 63 consecutive patients with severe COVID-19 pneumonia and 27 healthy subjects as control group. Clinical laboratory findings and plasma protein concentration of KKS peptides [bradykinin (BK), BK1-8], KKS proteins [high–molecular weight kininogen (HK)], and KKS enzymes [carboxypeptidase N subunit 1 (CPN1), kallikrein B1 (KLKB1), angiotensin converting enzyme 2 (ACE2), and C1 esterase inhibitor (C1INH)] were analyzed. We detected dysregulated KKS in patients with COVID-19, characterized by an accumulation of BK1-8 in combination with decreased levels of BK. Accumulated BK1-8 was related to severity of patients with COVID-19. A multivariate logistic regression model retained BK1-8, BK, and D-dimer as independent predictor factors to intensive care unit (ICU) admission. A Youden’s optimal cutoff value of −0.352 was found for the multivariate model score with an accuracy of 92.9%. Multivariate model score-high group presented an odds ratio for ICU admission of 260.0. BK1-8 was related to inflammation, coagulation, and lymphopenia. Our data suggest that BK1-8/BK plasma concentration in combination with D-dimer levels might be retained as independent predictors for ICU admission in patients with COVID-19. Moreover, we reported KKS dysregulation in patients with COVID-19, which was related to disease severity by means of inflammation, hypercoagulation, and lymphopenia.

## Introduction

COVID-19 pandemic has caused more than 5 million deaths (1) and, in severe cases, is characterized by multiple clinical manifestations ranging from lymphocytopenia to hyperinflammatory state, procoagulant disorder, and thrombotic events (2). The development of a “cytokine storm” has been widely studied and is directly related to the hyperinflammatory conditions (3). In addition, persistent and elevated inflammation is related to the development of thrombotic dysregulation (4). This thromboinflammatory disorder has been extensively reported and is implicated in severity and mortality risk (5). Patients with severe COVID-19 also develop strong dysregulation of vascular permeability (6), which is related to multiple clinical complications as pulmonary edema (7). Tight regulation of vascular permeability is essential for the maintenance of homeostasis, especially under inflammatory conditions that are a main cause of vascular alteration (8, 9). To ensure this regulation, there are two parallel mechanisms: the renin-angiotensin system (10) and the kallikrein-kinin system (KKS) (9, 11). Both systems are intrinsically connected and are essential to maintain vascular homeostasis and are as well implicated in inflammation and tissue repair (12). Connecting both mechanisms, we find the angiotensin converting enzyme 2 (ACE2), which is involved in the regulation of angiotensin peptides (12). In fact, ACE‐2 is directly related to COVID-19, as it is well-recognized to be involved in the entry of SARS-CoV-2 into host cells (13, 14). However, smaller attention has been given to the intricate associations of the KKS, which, as other authors have previously mentioned, could be of great importance in the context of COVID-19 severity (15–18).

Bradykinin (BK) formation and regulation are initiated by the activation of coagulation factor XII. Coagulation factor XII is able to convert prekallikrein to kallikrein (KLKB1), which, in turn, activates factor XII in a positive feedback loop that requires C1-esterase inhibitor (C1INH) to get inactivated (19). KLKB1 is involved in the processing of high–molecular weight kininogen (HK) into BK, which can bind BK receptor B2 (BDKRB2) (20). BDKRB2 is expressed in endothelial cells, and its activation is involved in vasodilatation and increased vascular permeability (21). However, BK is a short-lived peptide (21), and it is rapidly degraded by several plasma enzymes as ACE or aminopeptidase P (22). In addition, carboxypeptidase N1 (CPN1) is also able of degrade BK, forming an eight–amino acid peptide: des-arg-9-bradykinin (BK1-8) (20). BK1-8 has a longer life in plasma than BK and binds to BK receptor B1 (BDKRB1) (23). Furthermore, when activated by BK1-8, BDKRB1 is related to cytokine release, leading to a positive feedback loop of inflammation (24). BK1-8 degradation is essential to regain homeostasis; however, the main enzyme involved in this process, ACE2, is critically imbalance in patients with COVID-19 (25). Studying the relation of BK peptides and inflammation in the context of COVID-19 is tempting and, as other authors have previously appointed, KKS dysregulation could be another “storm” in the horizon of COVID-19 pathophysiology and severity (18, 26). Hence, we aim to assess the expression of KKS components as well as the relation of BK and BK1-8 with clinical prognosis and COVID-19 hyperinflammation and procoagulant state.

## Methods

### Study Subjects

We recruited 63 consecutive hospitalized patients with COVID-19 on the day 1 of hospital admission according to the following inclusion criteria: positive result in (RT-PCR) Real Time Polymerase Chain Reaction assay for SARS‐CoV-2; abnormalities or infiltrates on chest x-ray/CT scan; room-air oxygen saturation <92% or requirement of supplemental oxygen; and age >18 years. Exclusion criteria were as follows: COVID-19 symptoms 14 days before hospitalization; concomitant systemic fungal or bacterial infection; immunodeficiency or neutropenia; active neoplasm; current systemic autoimmune or auto-inflammatory disease; severe pulmonary disease requiring home oxygen therapy; and previous therapy with oral corticosteroids or anti-inflammatory cytokines.

Exploratory endpoints were 60-day mortality, ICU admission for intubation and mechanical ventilation, and duration of hospitalization. Twenty-seven healthy controls (HC) without evidence of respiratory or infectious disease were selected as control group.

The study was approved by local Ethics Committee (PI-4087), and informed consent was obtained from all participants.

### Plasma and Peripheral Blood Mononuclear Cell Isolation

Peripheral blood of 20 ml was collected using butterfly-winged needles with a needle size of 21G and 9-ml EDTA polypropylene tubes by venipuncture. In addition, blood collection was performed using the aspiration technique applying a constant move. Moreover, we have rapidly performed sampling of blood and avoided any time delays after venipuncture. Precisely, plasma isolation was performed within 30 min after blood collection by layering blood over 10-ml Ficoll-Paque Plus (Amersham Bioscience, Sweden) and centrifuging 2,000 rpm for 10 min at 21°C. Plasma was removed from the upper layer and peripheral blood mononuclear cells (PBMCs) were acquired from the interphase and washed two times in PBS. Plasma was stored at −80°C and freeze-thaw cycles were avoided. Finally, all the blood samples collected (ICU/non-ICU patients and HCs) were treated in an identical manner.

### Plasma Protein Concentration Analysis

Specific ELISA kits were used according to the manufacturer’s instructions as available in **Table S3** to measure specific plasma concentrations of KKS proteins [high–molecular weight kininogen (HK)], KKS peptides (BK, BK1-8), and KKS enzymes [carboxypeptidase N subunit 1 (CPN1), kallikrein B1 (KLKB1), C1 esterase inhibitor (C1INH), and ACE2]. Moreover, we have measured IL-1β, IL-6, TNFα, tissue factor (TF), CD40L, and gasdermin D (GSDMD). Measurements for plasma samples were performed in duplicate. In all cases, intra-assay variability was CV% < 8% and inter-assays variability was CV% < 15%.

### Cytokine Concentration Analysis

Inflammatory cytokines IL-1β, IL-6, and TNF-α concentrations were measured from supernatants of overnight-cultured isolated PBMCs from patients with COVID-19 or HCs. Cytokine quantification was performed using a BD Human Inflammatory Cytokine CBA kit (551811, Becton-Dickinson Biosciences, Belgium), acquired by BD FACS-Calibur flow cytometer (Becton-Dickinson Biosciences, Belgium) and analyzed by FCAP Array software (Becton-Dickinson Biosciences, Belgium).

### Western Blot

Plasmas from HC and COVID-19 were diluted with PBS 1:20. Then, plasma proteins were separated by 10% gradient SDS-PAGE gels (Bio-Rad, Madrid, Spain) under reducing conditions and blotted on nitrocellulose membranes. HK was immunostained with a polyclonal anti-human HK antibody developed in rabbit (ab226087, Cambridge, UK), followed by a monoclonal anti-rabbit (Immunoglobulin G- Horseradish peroxidase) IgG-HRP–linked antibody (A9452-1VL, Sigma-Aldrich, Madrid, Spain). Detection was done with enhanced chemiluminescence (ECLTM Prime; Amersham).

### Blood mRNA Isolation and BDKRB1 Quantification by qPCR

Total RNA was extracted from blood samples using TRIzol (TRI reagent) following the manufacturer’s protocol (ref: 10296028, Life Technologies, Canada). RNA levels were measured by RTqPCR using QuantiMix Easy kit (Biotools, Spain) and Light-Cycler system (Roche Diagnostics, Switzerland) and results normalized to 18S expression. Primer sequences:

18S: F: CGGCGACGACCCATTCGAAC and R: GAATCGAACCCTGATTCCCCGTC; BDKRB1: F: AGGCCAATTTGTTCATCAGC and R: AGGCCAGGATGTGGTAGTTG.

### Statistical Analysis

Data are presented as mean ± standard error mean (SEM). Comparisons were performed by Mann–Whitney U-test or chi-squared test. For quantitative variable correlation, Spearman’s rho analysis was performed. Receiver operating characteristic (ROC) analysis was achieved by Brown/Wilson test and to calculate optimal cutoff values Youden index was used. In all cases, level of significance (alpha) was set at 0.05. Analyses were performed using Prism 8.0 (Graph Pad, USA) and SPSS 26.0 (IBM, USA) software.

## Results

### Characteristics of the Study Subjects

Twenty-seven HC subjects and 63 severe COVID-19 (COV) pneumonia patients were recruited on the day 1 of hospital admission. HC and COV were homogeneous in sex (67 vs. 75% males, respectively), age (51 ± 14 vs. 54 ± 12 years, respectively), and body mass index (28.3 ± 5.4 vs. 29.2 ± 6.5 kg/m^2^, respectively). During the follow-up period, four patients died, 16 patients required mechanical ventilation, and 17 patients were admitted to ICU. Detailed clinical characteristics of patients with COVID-19 are shown in **Table 1**.

**Table 1:** General characteristics of the patients with COVID-19 pneumonia and healthy controls*.

	Patients With COVID-19	Healthy Controls	P-Value
Age, years ± SD	54 ± 12	51 ± 14	0.3044
Sex, male/female	47/16	18/9	0.4411
Body mass index, kg/m^2^	29.2 ± 6.5	28.3 ± 5.4	0.5293
Days since onset of symptoms	8.8 ± 3.6	NA	NA
Symptoms at admission, n (%)			
Cough	32 (50)	NA	NA
Active fever	32 (50)	NA	NA
Dyspnea	32 (50)	NA	NA
Myalgia	16 (25)	NA	NA
Sputum production	9 (14)	NA	NA
Chest tightness	2 (3)	NA	NA
Headache	11 (17)	NA	NA
Fatigue	13 (21)	NA	NA
Anorexia	4 (6)	NA	NA
Nausea	5 (8)	NA	NA
Diarrhea	13 (21)	NA	NA
Chest pain	7 (11)	NA	NA
Anosmia	6 (10)	NA	NA
Comorbidities, n (%)			
Hypertension	18 (29)	NA	NA
Coronary artery disease	4 (6)	NA	NA
Diabetes mellitus	12 (19)	NA	NA
Obesity	14 (22)	NA	NA
Chronic lung disease	9 (14)	NA	NA
Chronic kidney disease	1 (2)	NA	NA
Hypothyroidism	2 (3)	NA	NA
Smoking history, n (%)			
Current	37 (59)	4 (14)	<0.001
Former	10 (16)	1 (3)	<0.001
Never	16 (26)	22 (81)	0.10
Pneumonia severity scores			
CURB-65	0.67 ± 0.78	NA	NA
Fine risk class	2.19 ± 1.0	NA	NA
Laboratory findings			
PaO_2_, mmHg	65.4 ± 13.8	NA	NA
PaO_2_/FiO_2_ ratio	249.7 ± 102.4	NA	NA
PaCO_2_, mmHg	34.2 ± 6.5	NA	NA
White cell count, 10^3^ cells/µl	7.19 ± 4.07	NA	NA
Neutrophils, 10^3^ cells/µl	5.51 ± 3.32	NA	NA
Lymphocytes, 10^3^ cells/µl	1.10 ± 1.70	NA	NA
Monocytes, 10^3^ cells/µl	0.32 ± 0.16	NA	NA
Platelets, 10^3^ cells/µl	230 ± 75	NA	NA
Hemoglobin, g/dl	13.9 ± 1.5	NA	NA
C-reactive protein, mg/L	84.2 ± 71.8	NA	NA
Aspartate aminotransferase, U/L	45.4 ± 28.8	NA	NA
Alanine aminotransferase, IU/L	44.6 ± 32.3	NA	NA
ϒ-Glutamyltransferase, IU/L	91.3 ± 92.7	NA	NA
Bilirubin, µmol/L	0.53 ± 0.24	NA	NA
Albumin, g/L	4.3 ± 0.3	NA	NA
Ferritin, ng/ml	882.8 ± 791.8	NA	NA
Lactate dehydrogenase, U/L	307.1 ± 104.0	NA	NA
D-dimer, ng/ml	1107 ± 1461	NA	NA
Fibrinogen, mg/dl	734.4 ± 264.0	NA	NA
Evolution results			
Duration of hospital stay, days	19.5 ± 20.7	NA	NA
Requirement of mechanical ventilation, n (%)	16 (25.4)	NA	NA
ICU admission, n (%)	17 (27.0)	NA	NA
Exitus, n (%)	4 (6.3)	NA	NA

*SD, standard deviation; PaO_2_, oxygen arterial pressure; FiO_2_, fractional inspired oxygen; PaCO_2_, carbon dioxide arterial pressure; ICU, intensive care unit. NA, not available.

### Dysregulated Plasma Levels of KKS

**Figure 1:**
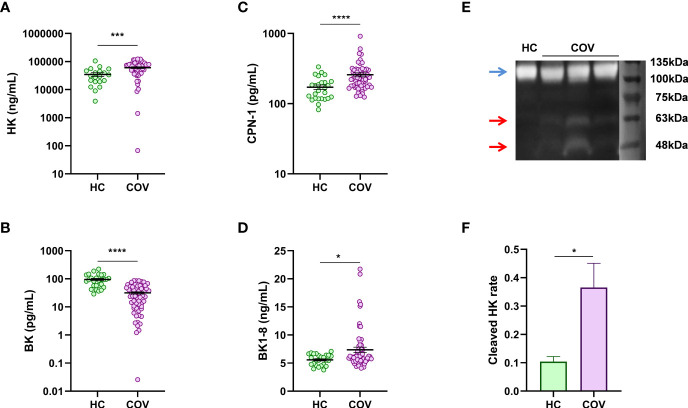
Plasma levels of KKS components. ELISA quantification in plasma from healthy controls (HC) and patients with COVID-19 (COV) of **(A)** HK (HC, n = 20; COV, n = 49), **(B)** BK (HC, n = 27; COV, n = 63), **(C)** BK1-8 (HC, n = 27; COV, n = 63), and **(D)** CPN1 (HC, n = 26; COV, n = 61). **(E)** Representative Western blot of plasma of healthy control (HC) and patients with COVID-19 patient (COV) (blue arrow, band at approximately 110 kDa is an intact 1-chain HMWK; red arrows, bands at approximately 46 and 56 kDa are cleaved 2-chain HMWK). **(F)** Cleaved 2-chain HK expression relative to intact-1chain HK expression in plasma from healthy controls (HC, n = 4) and patients with COVID-19 (COV, n = 8) measured by densitometry quantification of bands from Western blot. Mean differences were analyzed by Mann–Whitney U-test. Error bars: mean ± SEM. *P < 0.05; ***P < 0.001; ****P < 0.0001.

In comparison with HC, patients with COVID-19 presented elevated plasma concentrations of HK (**Figure 1A**), a compound that is delivered to the blood by the liver under basal conditions and that could be released by other tissues, such as alveoli epithelium, under inflammatory conditions (27). However, BK plasma levels were reduced in patients with COVID-19 (**Figure 1B**), which may suggest an impairment of its production or an increase of its degradation. We measured the plasma concentration of KLKB1, the enzyme involved in BK release, and the observed normal levels compared to HC (**Figure S1A**). Interestingly, altered levels of BK might be implicated in COVID-19 coagulation complications. Moreover, C1INH that is involved in KLKB1 regulation also presented similar levels in patients with COVID-19 and HC (**Figure S1B**). Regarding BK degradation toward smaller peptides, we observed high plasma levels of its byproduct: BK1-8 (**Figure 1C**). Concomitantly, CPN1, the major BK degrading enzyme, was upregulated in patients with COVID-19 (**Figure 1D**). To further address KKS activation in patients with COVID-19, we performed HK Western blot, and we observed nearly threefold increase in relative level of HK proteolytically cleaved forms in plasma from patients with COVID-19 compared to healthy controls (**Figures 1E, F**), which could confirm contact pathway activation. These consecutive results suggest that KKS is critically dysregulated in patients with COVID-19, promoting the formation of active peptide BK1-8, thus reducing BK. BK1-8 accumulation must be resolved by ACE2; however, this enzyme is slightly but significantly reduced in plasma from patients with COVID-19 (**Figure S1C**) as other authors have previously described (25). In addition, BK1-8 main receptor, BDKRB1, mRNA expression were upregulated in circulating cells from patients with COVID-19 (**Figure S1D**).

### Clinical Relevance of BK1-8/BK Plasma Concentrations in Patients With Severe COVID-19

**Figure 2:**
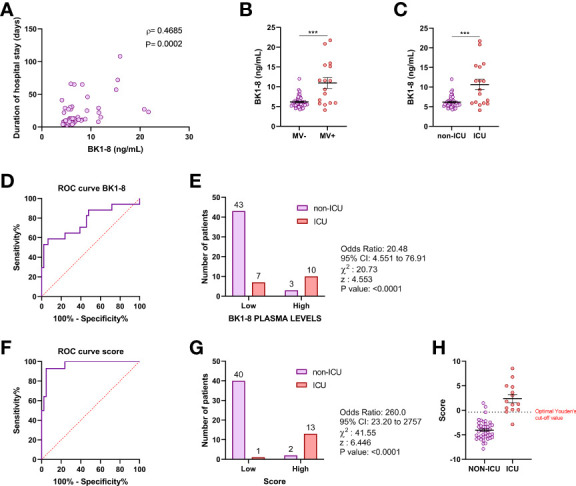
BK1-8 association with COVID-19 severity. **(A)** Correlation of BK1-8 plasma concentration and duration of hospital stay (n = 60). Spearman’s correlation coefficient (ρ) and P‐value are shown. **(B)** Comparison of BK1-8 plasma concentration in patients not requiring mechanical ventilation (MV−, n = 47) and those requiring it (MV+, n = 16). **(C)** Comparison of BK1-8 plasma concentration in patients not derived to ICU (non-ICU, n = 46) and patients derived to ICU (ICU, n = 17). Mean differences were analyzed by Mann–Whitney U-test. Error bars: mean ± SEM. ***: *P* < 0.001. **(D)** Receiver operating characteristic (ROC) curve for predictive performance value for ICU admission of BK1-8 (n = 63). **(E)** Contingency table comparing ICU admission for patients with high and low BK1-8 plasma levels. **(F)** ROC curve for predictive performance value for ICU admission of multivariate model score (n = 56). **(G)** Contingency table comparing ICU admission for patients with high and low multivariate model score. ROC curves were analyzed by Wilson/Brown test. Contingency tables were analyzed by chi-squared test. **(H)** Multivariate model score in patients with COVID-19 not derived to ICU (non-ICU, n = 42) and derived to ICU (ICU, n = 14).

In patients with COVID-19, plasma levels of BK1-8 were associated with duration of hospital stay (ρ = 0.469; *P* = 0.0002) (**Figure 2A**) and were elevated in patients requiring mechanical ventilation (**Figure 2B**) or ICU admission (**Figure 2C**). We performed a ROC curve analysis of BK1-8 levels for ICU admission (**Figure 2D**) and found an optimal Youden’s cutoff value of 8.201 ng/ml (**Table S1**). Contingency table showed an odds ratio of 20.48 for ICU admission in the high BK1-8 plasma level group (**Figure 2E**). In a logistic regression model, D-dimer level—previously described as biomarker of COVID-19 severity (28)—BK1-8 and BK levels were retained as independent predictors of ICU admission (**Table S2**). ROC curve for the multivariate model score was very efficient with a Youden’s cutoff value of −0.3521 (sensitivity, 92.86%; specificity, 95.24%) (**Figure 2F**), being more accurate than any of the parameters alone (**Table S2**). Contingency table showed an odds ratio of 260.0 for ICU admission in the high-score group (**Figure 2G**). Hence, we present BK/BK1-8 plasma concentrations as potentially valuable markers for COVID-19 severity which, when combined in a model with D-dimer, yield an accuracy of 92.9% for ICU admission in our cohort (**Figure 2H**).

### BK1-8 Dysregulation Is Related to Hyperinflammatory and Prothrombotic State of Patients With COVID-19

**Figure 3:**
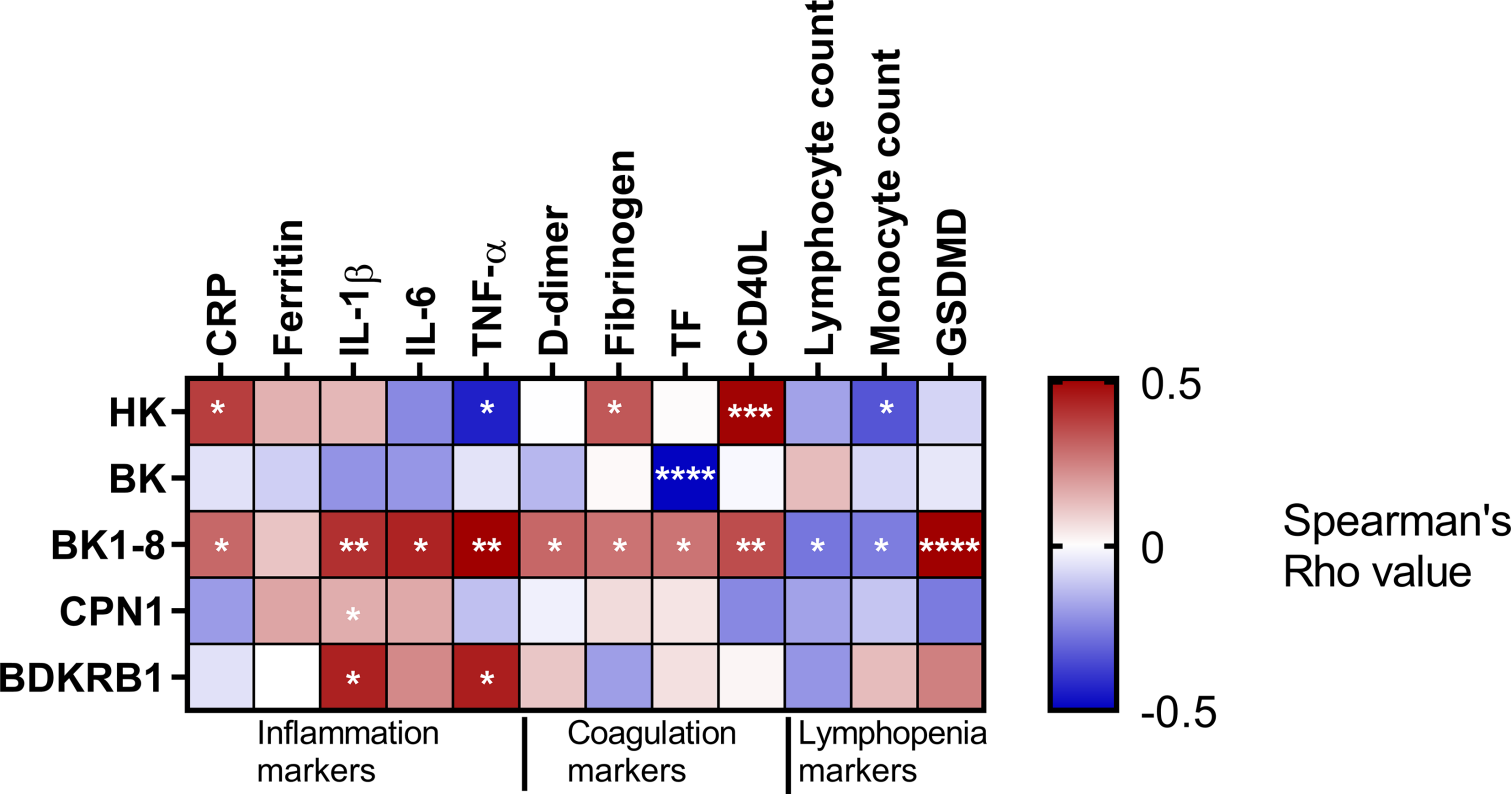
KKS association with inflammation, coagulation, and lymphopenia. Heatmap representing Spearman’s correlation coefficients between BK cascade components and inflammation, coagulation, or lymphopenia markers. HK, high–molecular weight kininogen; BK, bradykinin; BK1-8, bradykinin1-8; CPN1 carboxypeptidase N subunit 1; BDKRB1, bradykinin receptor B1; CRP, C-reactive protein; IL, interleukin; TNF, tumor necrosis factor; TF, tissue factor, GSDMD, Gasdermin D. Significant associations are marked by asterisks. *P < 0.05; **P < 0.01; ***P < 0.001; ****P < 0.0001.

Regarding the hyperinflammation, we observed BK1-8 was positively and significantly related to four out of five inflammation markers studied (CRP, ferritin, IL-1β, IL-6, and TNF-α) (**Figure 3**), suggesting a potential role of this peptide in patients with COVID-19’ inflammation. Meanwhile, the prothrombotic state markers such as fibrinogen and CD40L were related to HK (**Figure 3**). In line with this, D-dimer, fibrinogen, tissue factor (TF), and CD40L were also directly related to BK1-8 (**Figure 3**).

Interestingly, the NLRP3 inflammasome plays a role in processing and release of the inflammatory cytokine IL-1β (29). Consistently, our data of NLRP3 showed a positive correlation with IL-1β (**Figure S2**), as suggested by previous studies where NLRP3 role in COVID-19 is highlighted (30–33).

Lastly, lymphopenia markers such as lymphocyte and monocyte counts negatively correlated with BK1-8. In line with this, the pyroptotic marker, GSDMD, showed a positive correlation with BK1-8, suggesting an implication of KKS in lymphopenia (**Figure 3**).

## Discussion

Our study of BK cascade suggested an impaired KKS in severity of patients with COVID-19. This regulation was characterized by high levels of HK and BK1-8 in combination with low levels of BK, all of these are part of contact system involved in intrinsic coagulation cascade activation (34). A reduction of BK levels could result in endothelial dysfunction because BK activation of BDKRB2 induces the release of nitrogen oxide, prostacyclin, endothelium-derived hyperpolarizing factor, and tissue plasminogen activator, which exert diverse physiological actions on the cardiovascular system, including regulation of vascular tone and local blood flow to organs, coagulation, fibrinolysis, and water-electrolyte balance (35). Conversely, high levels of BK could lead to capillary leakage and thus angioedema, which also constitutes a COVID-19 complication. In this line, blocking BDKRB2 and inhibiting KLKB1 activity have been proposed to ameliorate early disease caused by COVID-19 (15, 18). BK plasma levels are regulated by several enzymes including CPN1, which cleaves BK to BK1-8 (36). In patients with COVID-19, we observed an overexpression of plasma CPN1, which we suggest collaborates in BK1-8 accumulation. It is interesting to further explore the role of CPN1 in COVID-19 pathophysiology as this carboxypeptidase also inactivates the complement system (37). Recently, CPN1 has been described as a promising biomarker for chemotherapeutic surveillance (38). In addition, our results from HK Western blot suggest that BK1-8 levels in plasma of patients with COVID-19 could not only result because of the increased level of CPN1 but also by an increased e production of kinins, *via* increased degradation of HK. Once BK1-8 is released to the plasma, it exerts multiple functions mainly by binding to BDKRB1, which stimulates cytokine release by macrophages and monocytes (24, 39, 40). Interestingly, inflammatory condition of patients with COVID-19 has been related to their prothrombotic state (41).

Interestingly, BDKRB1 receptor is expressed in the endothelium and in leukocytes under inflammatory conditions as those expected in patients with COVID-19 (42). Indeed, Nicolau et al. hypothesized that targeting BDKRB1 pathway may be beneficial in SARS-CoV-2 infection (17). BK1-8 accumulation is at the basis of several pathophysiological conditions (43–45), and some authors have hypothesized about its role in COVID-19 progression (26), so we were tempted to study its relation with clinical markers for COVID-19 severity. In our study, BK1-8 is related to COVID-19 severity; indeed, we propose BK and BK1-8 when combined in a model with D-dimer plasma concentrations as potentially valuable markers for COVID-19 severity.

We observed that BK1-8 was positively and significantly related to inflammation, suggesting a potential role of this peptide in inflammation in patients with COVID-19. Interestingly, inflammatory condition of patients with COVID-19 has been related to NLRP3 inflammasome (30–33). Further study of the KKS is quite interesting, especially in the context of thromboinflammation and pulmonary edema complications; in fact, therapeutical targeting on this pathway has been addressed (15). BK1-8 was also directly related to coagulation markers: D-dimer, fibrinogen, tissue factor (TF), and CD40L, although the mechanistic relation was not addressed in this study. In contrast, BK was negatively related with TF; indeed, it has been described that BK can inhibit TF expression (46). We hypothesize that hyperinflammatory state and damaged endothelial function (47), both related to KKS (48), might be associated with patients with COVID-19 prothrombotic complications, although a more detailed evaluation of this process is needed. Finally, we observed a relation between BK1-8 concentration and lymphopenia assessed by lymphocyte and monocyte count and GSDMD plasma concentration. These findings suggest that lymphopenic states in patients with COVID-19 along with damaged endothelial function (49, 50) are facilitating the accumulation of BK1-8, as lymphocytes and monocytes, as well as endothelial cells, are the main source of ACE2, the BK1-8‐degrading enzyme (51).

This study supports with patients’ data the suggested importance of the BK peptides in severe patients with COVID-19, which we propose are linked to ICU admission. Interestingly, Garvin and colleagues reported altered KKS in bronchoalveolar lavage fluid (BALF) of patients with COVID-19, showing reduced levels of ACE in combination with enhanced ACE2 and kininogen expression. In addition, authors highlighted overexpression of kallikrein enzymes and both BK receptors (12). In contrast, our data showed low levels of ACE2 plasma protein expression; this disagreement might be due to the difference of biological samples (BALF vs. plasma). Interestingly, other studies have shown elevated ACE2 in postmortem lung samples (13). On the other side, Lipcsey and colleagues reported evidence of activation of KKS and complement in 66 critically ill patients with COVID-19. Indeed, this activation was related with the clinical outcome (14). In line with this, our data also suggest that the KKS dysregulation in patients with COVID-19 is potentially associated with COVID-19 severity.

Our study has several limitations, which we recognize. First, we did not used specific tubes containing precise protease inhibitors for KKS assessment. Contact activation could alter HK, BK, and BK1-8 concentrations if samples are not adequately stabilized immediately at the time of blood collection. Second, healthy controls cohort information was limited. Third, limited sample size and lack of follow-up time restrained the potential identification of robust prognostic events. Fourth, this is an observational study carried out in patients with severe COVID-19 pneumonia treated according to conventional clinical practice, so the non-randomization does not allow us to infer the efficacy of different clinical approaches.

Altogether, we have observed that KKS is dysregulated in patients with COVID-19. Interestingly, the most relevant finding of this study is the relation of soluble plasma concentrations of BK1-8 and BK with COVID-19 severity, suggesting these two kinin components as possible biomarkers of COVID-19 severity and additional study of this possibility is promising. BK1-8 was associated to COVID-19 hyperinflammatory and prothrombotic state as well as lymphopenia. However, we acknowledge that the mechanistic and functional relation of BK1-8 and COVID-19 pathophysiology remains mostly unclear and must be further addressed. Our results contribute to the rising interest in KKS signaling in patients with COVID-19 due to its implication in thromboinflammation and pulmonary edema complications.

## Data Availability Statement

The datasets presented in this study can be found in online repositories. The names of the repository/repositories and accession number(s) can be found in the article/**Supplementary Material**.

## Ethics Statement

The studies involving human participants were reviewed and approved by the Ethics committee from La Paz University Hospital with number PI-4087. The patients/participants provided their written informed consent to participate in this study.

## Author Contributions

FG-R and CC-Z conceptualized the study; ED-G, EZ, AM, FG-R, and CC-Z advised on the study design and endpoints; ED-G, SG-T, EA, RP-d-D, KN-N, FG-R, and CC-Z performed designed experiments; EZ, AM, and RG recruited patients with COVID-19 and collect samples; ED-G, EA, EL-C, FG-R, and CC-Z analyzed data and performed statistical data; FG-R and CC-Z were responsible for the study management and coordination; FG-R and CC-Z drafted the paper. All authors contributed to the article and approved the submitted version.

## Funding

This research was funded by Health Research Fund (Fondo de Investigación Sanitario [FIS])-European Regional Development Fund (FEDER), Spain, through PI19/01612 (FG-R) and COV20/00207 and PI19-01363 (CC-Z) and ISCIII (CP18/00028), co-funded by ESF, “Investing in your future”.

## Conflict of Interest

The authors declare that the research was conducted in the absence of any commercial or financial relationships that could be construed as a potential conflict of interest.

## Publisher’s Note

All claims expressed in this article are solely those of the authors and do not necessarily represent those of their affiliated organizations, or those of the publisher, the editors and the reviewers. Any product that may be evaluated in this article, or claim that may be made by its manufacturer, is not guaranteed or endorsed by the publisher.
