# Supplementary material for: Impaired Kallikrein-Kinin System in COVID-19 Patients' Severity
Source: Front Immunol. 2022 Jun 22;13:909342. doi: 10.3389/fimmu.2022.909342 (PMC9258198; doi:10.3389/fimmu.2022.909342)
Supplement: Supplementary file 1 [file DataSheet_1.pdf]

**Supplemental Material**

**Tables**

**Table S1**. Receiving Operating Characteristic (ROC) curve analysis by Brown–Wilson test for ICU admission in patients with severe COVID-19 pneumonia using isolated values of BK1-8, BK and D-dimer and the multivariate model score

| **Variables** | **ROC analysis by Brown-Wilson Test** | | | |
|---|---|---|---|---|
| | **A.U.C.** | **95% C.I.** | ***P*-value** | **Youden cut-off value (sensitivity, specificity)** |
| **BK1-8** | 0.770 | 0.620 to 0.920 | 0.0011 | 8.201 ng/mL (58.8%, 93.48%) |
| **BK** | 0.734 | 0.610 to 0.858 | 0.0047 | 31.17 pg/mL (88.24%, 60.0%) |
| **D-dimer** | 0.840 | 0.719 to 0.961 | 0.0002 | 1425 ng/mL (71.4%, 90.5%) |
| **Multivariate model score** | 0.966 | 0.922 to 1.000 | <0.0001 | -0,3521 (92.9%, 95.2%) |

A.U.C.: area under the curve, C.I.: confidence interval. *P*-value and Youden optimal cut-off value (including sensitivity and specificity) are shown.

**Table S2** Multivariate logistic regression model including BK1-8, BK and D-dimer as independent determinants of ICU admission in patients with severe COVID-19 pneumonia

| | **B** | **S.E.** | **Wald** | **p-Value** | **Exp(B)** | **95.0% C.I. for EXP(B)** | |
|---|---|---|---|---|---|---|---|
| | | | | | | Lower | Upper |
| **BK1-8** | 0.709 | 2.52 | 7.913 | 0.005 | 2.032 | 1.240 | 3.329 |
| **BK** | -0.044 | 0.045 | 0.919 | 0.338 | 0.957 | 0.876 | 1.047 |
| **D-dimer** | 0.001 | 0.000 | 6.353 | 0.012 | 1.001 | 1.000 | 1.002 |
| **Constant** | -7.444 | 2.376 | 9.821 | 0.002 | 0.001 | | |

S.E.: standard error, C.I.: confidence interval.

**Table S3**. ELISA kits used in the study

| ELISA target | Manufacturer | Reference | Detection limit |
|---|---|---|---|
| **HK** | CUSABIO technology, USA | CSB-EL012479HU | 47 ng/mL |
| **BK** | Cloud-Clone Corp., USA | CEA874Hu | 0.51 pg/mL |
| **BK1-8** | MyBioSource, USA | MBS109439 | 0.1 ng/mL |
| **CPN1** | Cloud-Clone Corp., USA | SEF323Hu | 6.7 pg/mL |
| **KLKB1** | FineTest, China | EH14758 | 0.375 ng/mL |
| **C1INH** | FineTest, China | EH2726 | 1.875 ng/mL |
| **ACE2** | Cloud-Clone Corp., USA | SEB886Hu | 5.5 pg/mL |
| **TF** | CUSABIO technology, USA | CSB-E07913h | 3.12 pg/mL |
| **CD40L** | Invitrogen, Austria | BMS293 | 0.06 ng/mL |
| **GSDMD** | MyBioSource, USA | MBS2705515 | 0.312 ng/mL |

## Figures

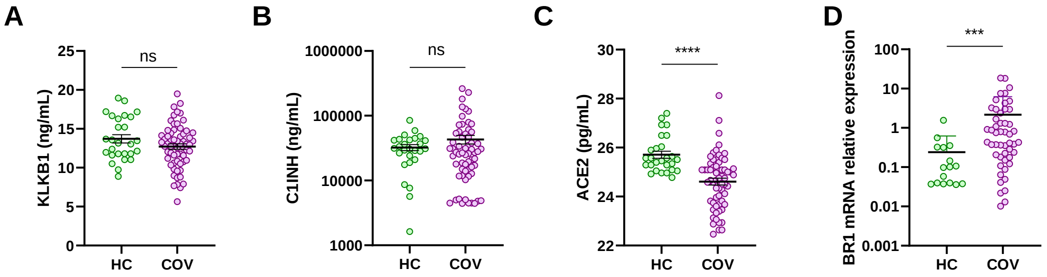


**Supplemental Figure S1. Kallikrein-kinin proteins quantification in plasma. (A)** ELISA quantification of KLKB1 concentration in plasma from HC (n=27) and COVID-19 patients (N=63). **(B)** ELISA quantification of C1INH concentration in plasma from HC (n=27) and COVID-19 patients (N=62). **(C)** ELISA quantification of ACE2 concentration in plasma from HC (n=27) and COVID-19 patients (N=63). **(D)** Relative BR1 mRNA expression in circulating cells from HC (n=16) and COVID-19 patients (n=50). Mean differences were analysed by Mann-Whitney U test. Error bars: mean ± SEM. ns: non-significant; ***: $P<0.001$; ****: $P<0.0001$.

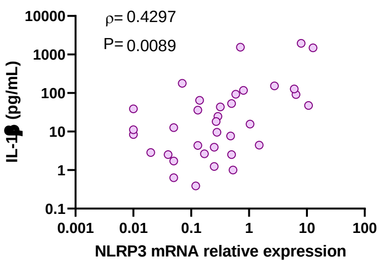


**Supplemental Figure S2**. **NLRP3 correlates with IL-1β**. Correlation of NLRP3 mRNA expression in PBMCs and IL-1β plasma concentration from COVID-19 patients (n=36). Spearman's correlation coefficient (ρ) and P-value are shown.
